# Network pharmacology and molecular docking-based study on exploring the potential mechanism of *Lycium barbarum* L: In the treatment of atherosclerosis

**DOI:** 10.1097/MD.0000000000035734

**Published:** 2023-11-03

**Authors:** Xinchen Qin, Zikai Xie, Xi Chen, Xiaoxuan Wang, Lijuan Ma

**Affiliations:** a Dr. Neher’s Biophysics Laboratory for Innovative Drug Discovery, State Key Laboratory of Quality Research in Chinese Medicine, Macau University of Science and Technology, Macau, China; b Independent Researcher, Zhuhai, China; c Guangdong Second Provincial General Hospital, Postdoctoral Research Station of Basic Medicine, School of Medicine, Jinan University, Guangzhou, China.

**Keywords:** atherosclerosis, Goji berries, molecular docking, network pharmacology

## Abstract

**Background:**

Goji berries (*Lycium barbarum* L) are herbal medicine that have a long history of use and multiple pharmacological activities. In this study, we investigated the potential therapeutic effects of Goji berries on atherosclerosis (AS) using network pharmacology and molecular docking.

**Methods:**

The active compounds of Goji berries were identified using the Traditional Chinese Medicine Systems Pharmacology platform, as well as the literature and the targets of each active compound were obtained using the Swiss Target Prediction database. The AS-related targets were collected from the GeneCards and OMIM databases to obtain the common targets of Goji berries and AS. The drug-compound-target-disease network and protein-protein interaction network were constructed using the Cytoscape software to obtain the core target proteins of Goji berries related to AS. Gene ontology analysis of the core targets and Kyoto encyclopedia of genes and genomes pathway enrichment analysis were performed by Metascape. The target-chemical correlations were verified using AutoDock molecular docking.

**Results:**

After analysis, 44 active compounds within Goji berries were obtained that exhibit associations with AS. Among these, the proteins exhibiting the highest degrees of interaction within the compound-targeted protein protein-protein interaction network were AKT1, SRC, MAPK3, MAPK1, RELA, and STAT3. The gene ontology-biology process analysis showed that compound-targeted proteins were mainly involved in regulating small molecule metabolic process, cellular response to chemical stress, reactive oxygen species metabolic process, and regulation of inflammatory response. Kyoto encyclopedia of genes and genomes pathway mainly included lipid and AS in which AKT1, SRC, MAPK3, and MAPK1 were involved. Advanced glycation end-product-receptor for advanced glycation end-product signaling pathway in diabetic complications, Chagas disease, and pancreatic disease. Molecular docking assessment showed that fucosterol is bound to AKT1, MAPK3, and SRC.

**Conclusion:**

This study demonstrates that network pharmacology and molecular docking analyses contribute to a better understanding of Goji berries active compounds and targets as potential therapeutic drugs for treating AS.

## 1. Introduction

Atherosclerosis (AS) stands as a chronic cardiovascular disease (CVD) primarily driven by lipid deposition within arterial walls.^[1]^ Inflammation plays a vital role in both physiological and pathological alterations throughout the progression of AS.^[2]^ While often associated with cardiac implications, AS can affect arteries across the body. AS may cause angina pectoris, myocardial infarction, arrhythmia, and even sudden death within the coronary arteries. Similarly, cerebral AS can trigger cerebral ischemia, brain atrophy, or stroke. Renal AS is often associated with nocturia, hypertension, or impairments in renal function. Additionally, when AS occurs in the extremities, it may lead to claudication or induce lowered blood pressure within the affected limbs.^[3,4]^ Generally, mild AS tends not to engender complications.

Traditional Chinese medicine (TCM) is progressively garnering recognition beyond Chinese regions due to its notable efficacy and minimal toxicity. Goji berries, the dried fruit of *Lycium barbarum* L., can be found in complex herbal formulations. Meanwhile, it is also used as a nutritional supplement to meals.^[5,6]^ Goji berries has the effect of nourishing the kidney and liver, contributing to enhance immunity, anti-aging effects, blood sugar modulation, and lipid reduction.^[7]^ Nutritional science is now increasingly emphasized for its crucial role in intervening against non-communicable diseases, with dietary management being more feasible and adherable for patients than other treatments. In the last decades, epidemiological, clinical, and experimental studies have shown that diet plays a central role preventing AS.^[8,9]^ Based on the established clinical utilization of Goji berries in TCM treatment, we raised the question of whether Goji berries could be used as a nutritional supplement to aid in the prevention and treatment of AS.

Due to the complex composition of Goji berries, it isn’t easy to systematically study its molecular pharmacological mechanism. Network pharmacology is an emerging discipline based on the theory of system biology, pharmacokinetic and pharmacodynamic properties of drugs, drug targets, and their pharmacological functions, analysis of biological system networks, and selection of specific signaling nodes for drug design and modification.^[10,11]^ In recent years, network pharmacology has been increasingly applied and recognized in the study of TCM.^[12]^

**Figure 1.**
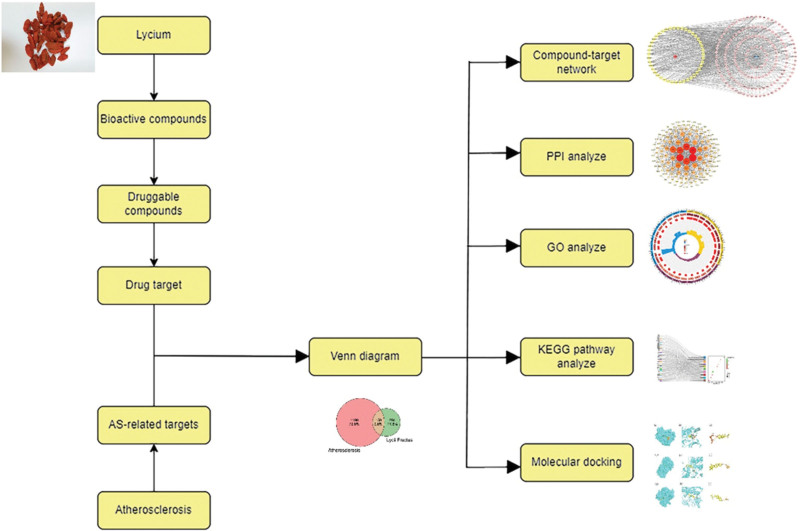
Schematic representation of the study workflow. The bioactive compounds of Goji berries were first identified by screening the traditional Chinese medicine systems pharmacology (TCMSP) and literature, and then AS-related genes were obtained by the Genecards and OMIM databases. The 2 gene sets were intersected to obtain 159 genes. Following functional enrichment analysis and protein-protein interaction (PPI) network construction, 6 core genes were filtered out and subjected to molecular docking and experimental validation.

In this study, network pharmacology was used to investigate the relationship between the bioactive components of Goji berries and AS-related target proteins. The pathway enrichment analysis and protein-protein interaction (PPI) analysis were used to derive the AS -related target proteins that Goji berries active compounds can interact with. Finally, the binding of Goji berries bioactive compounds to AS core target proteins was verified by molecular docking assay (Fig. 1). Our results provide theoretical support for the use of Goji berries in the prevention and treatment of AS.

## 2. Methods

### 2.1. Screening of bioactive compounds of Goji berries

In the traditional Chinese medicine systems pharmacology, (https://old.tcmsp-e.com/tcmsp.php),^[13]^ the chemical composition of Goji berries was searched and the components were screened according to OB (oral bioavailability) ≥30%^[14]^ and DL (drug-likeness) ≥0.18.^[15]^

### 2.2. Active-compound targets prediction

SMILES (simplified molecular input line entry specification) was obtained by searching for each active compound in “2.1” through the compound database PubChem (https://pubchem.ncbi.nlm.nih.gov).^[16]^ Then, the SMILES of each chemical was entered into the Swiss Target Prediction (http://www.swisstargetprediction.ch/index.php) database,^[17]^ and the species was set as *Homo sapiens* to obtain the predicted target of the compound. All results were collected and pooled to get the action targets of Goji berries.

### 2.3. AS-related target proteins screening

AS-related target proteins were searched in the human gene database GeneCards (https://www.genecards.org)^[18]^ and the online Mendelian human genetic database OMIM (http://www.omim.org).^[19]^ The outcomes of these searches were then combined and summarized to obtain AS-related disease target proteins.

### 2.4. Drug-component-target-disease network construction

The AS-related target proteins and Goji berries active-compound targets were imported into the target standardization database UniProt (https://www.uniprot.org),^[20]^ transformed into standard gene names, and imported into Cytoscape software (http://www.cytoscape.org),^[21]^ construct the drug-component-target-disease network. The resultant network was subsequently subjected to a node degree analysis, informing the classification of nodes via diverse colors and shapes.

### 2.5. PPI (protein-protein interaction) network construction

The potential targets of Goji berries acting on AS were obtained by importing the amalgamated compilation of Goji berries component targets and AS targets into Venny 2.1.0 software and taking the intersection of the 2 datasets. Subsequently, the potential targets of Goji berries in the context of AS, derived from Venny analysis, were introduced to the STRING 11.5 analysis platform (https://cn.string-db.org),^[22]^ “Multiple Proteins” was selected, species was set to *Homo sapiens*, confidence of screening condition was set to highest confidence (≥0.9), obtain the PPI network, and save its related TSV format information file. The TSV files were imported into Cytoscape, organized and plotted to obtain the PPI network graph. Then, the network topology analysis was performed, with the node color intensity and size indicative of high or low degree. While edge thickness corresponded to the combined score between interconnected nodes.

### 2.6. GO (gene ontology) and KEGG (Kyoto encyclopedia of genes and genomes) pathway analysis

The potential targets of Goji berries acting on AS were entered into the Metascape database (https://metascape.org),^[23]^ the species was selected as *Homo sapiens*, and KEGG analysis and GO enrichment analysis were performed, respectively. Outcomes were subsequently filtered based on a significance threshold of *P* < .05, ranked in ascending order according to *P* values, and the top 10 entries with the smallest *P* values were selected, and they were visualized using online tools (http://www.bioinformatics.com.cn/).

### 2.7. Molecular docking

To elucidate the binding interactions, molecular docking assays were conducted involving the core target proteins obtained and the primary active compounds present in Goji berries using AutoDockTools software.^[24]^ The 3D structures of the key active compounds of Goji berries, including fucosterol, betaine, and quercetin, were obtained from the PubChem database. The subsequent conversion of their 3D structure files into PDB format using OpenBabelGUI software.^[25]^ The converted 3D structures were imported into AutoDockTools, optimized, and saved as molecularly docked ligands. 3D structures of AKT1, SRC, MAPK3, MAPK1, RELA and STAT3 were downloaded from the PDB database (https://www.rcsb.org).^[26]^ AKT1 (PDBID: 7NH5, resolution: 1.90 Å), SRC (PDBID: 1FMK, resolution: 1.5 Å), MAPK3 (PDBID: 4QTB, resolution: 1.40 Å), MAPK1 (PDBID: 4QTA, resolution: 1.45 Å), RELA (PDBID: 6NV2, resolution: 1.130 Å), and STAT3 (PDBID: 6NJS, resolution: 2.7 Å) for energy minimization and imported into AutoDockTools software after removing impurities using Pymol software.^[27]^ Water molecules were removed, non-polar hydrogen atoms were added, and charges were calculated and saved as molecular docking receptors. The resulting receptor was molecularly docked with the ligand, and the number of dockings was set to 50 to obtain the final docking score. The lower the score, the lower the binding energy, the easier the receptor and ligand can bind, and the more stable the binding configuration.

## 3. Results

### 3.1. The active compounds of Goji berries

The components of Goji berries were searched by the traditional Chinese medicine systems pharmacology platform and screened according to OB ≥30% as well as DL ≥0.18 as conditions, and a total of 44 compounds were obtained, as shown in Table 1. Molecules with DL ≥0.18 are considered “drug-like” compounds,^[28]^ with higher OB and DL having a higher druggability. These include quercetin, betaine, atropine, β-sitosterol, fucosterol, stigmasterol, etc.

**Table 1:** Active compounds of Goji berries.

ID	NAME	MW	OB	DL
MOL001323	Sitosterol alpha1	426.8	43.28	0.78
MOL003578	Cycloartenol	426.8	38.69	0.78
MOL001979	LAN	426.8	42.12	0.75
MOL000449	Stigmasterol	412.77	43.83	0.76
MOL000358	Beta-sitosterol	414.79	36.91	0.75
MOL005438	Campesterol	400.76	37.58	0.71
MOL006209	Cyanin	411.66	47.42	0.76
MOL007449	24-methylidenelophenol	412.77	44.19	0.75
MOL008173	Daucosterol_qt	414.79	36.91	0.75
MOL008400	Glycitein	284.28	50.48	0.24
MOL010234	Delta-Carotene	536.96	31.8	0.55
MOL000953	CLR	386.73	37.87	0.68
MOL009604	14b-pregnane	288.57	34.78	0.34
MOL009612	(24R)-4alpha-Methyl-24-ethylcholesta-7,25-dien-3beta-ylacetate	482.87	46.36	0.84
MOL009615	24-Methylenecycloartan-3beta,21-diol	456.83	37.32	0.8
MOL009617	24-ethylcholest-22-enol	414.79	37.09	0.75
MOL009618	24-ethylcholesta-5,22-dienol	412.77	43.83	0.76
MOL009620	24-methyl-31-norlanost-9(11)-enol	428.82	38	0.75
MOL009621	24-methylenelanost-8-enol	440.83	42.37	0.77
MOL009622	Fucosterol	412.77	43.78	0.76
MOL009631	31-Norcyclolaudenol	440.83	38.68	0.81
MOL009633	31-norlanost-9(11)-enol	414.79	38.35	0.72
MOL009634	31-norlanosterol	412.77	42.2	0.73
MOL009635	4,24-methyllophenol	414.79	37.83	0.75
MOL009639	Lophenol	400.76	38.13	0.71
MOL009640	4alpha,14alpha,24-trimethylcholesta-8,24-dienol	426.8	38.91	0.76
MOL009641	4alpha,24-dimethylcholesta-7,24-dienol	412.77	42.65	0.75
MOL009642	4alpha-methyl-24-ethylcholesta-7,24-dienol	426.8	42.3	0.78
MOL009644	6-Fluoroindole-7-Dehydrocholesterol	402.7	43.73	0.72
MOL009646	7-O-Methylluteolin-6-C-beta-glucoside_qt	318.3	40.77	0.3
MOL009651	Cryptoxanthin monoepoxide	568.96	46.95	0.56
MOL009653	Cycloeucalenol	426.8	39.73	0.79
MOL009660	ipolamiide	406.43	39.43	0.47
MOL009662	Lantadene A	552.87	38.68	0.57
MOL009664	Physalin A	526.58	91.71	0.27
MOL009665	Physcion-8-O-beta-D-gentiobioside	608.6	43.9	0.62
MOL009677	lanost-8-en-3beta-ol	428.82	34.23	0.74
MOL009678	lanost-8-enol	428.82	34.23	0.74
MOL009681	Obtusifoliol	426.8	42.55	0.76
MOL000098	quercetin	302.25	46.43	0.28
MOL000430	betaine	117.17	40.92	0.01
MOL009650	Atropine	289.41	42.16	0.19
MOL009659	Hypaconitine	615.79	7.16	0.26

DL = drug-likeness, OB = oral bioavailability.

### 3.2. Potential targets of Goji berries acting on AS

**Figure 2.**
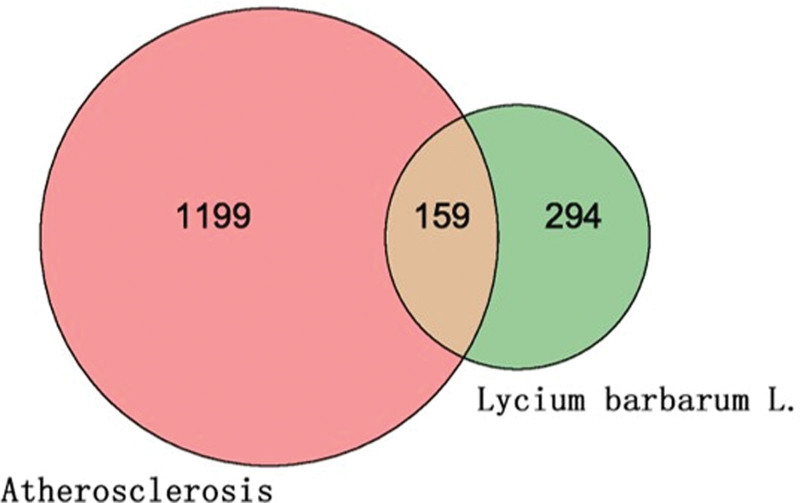
Overlapping target proteins between Goji berries and AS. 453 target proteins were associated with Goji berries, and 1358 proteins were related to AS. The intersection yielded 159 proteins shared by both Goji berries and AS.

The SMILES of 44 major components of Goji berries were found in PubChem and were entered into the Swiss Target Prediction database to predict the potential action targets. After sorting and removing duplicate targets, the 44 active compounds of Goji berries yielded 453 action targets. The targets were searched in the GeneCards OMIM databases with “AS” as the keyword, and the relevance score threshold was set to surpass the average. The genes obtained from the GeneCards were 1243, and an additional 119 genes were from the OMIM database. These datasets were sorted and merged to obtain 1358 targets. The AS-related targets and Goji berries active-compound targets were imported into Venny 2.1.0 software to draw a Venn diagram (Fig. 2). The overlap between these sets was taken as the potential targets of Goji berries in AS, with a total of 159 targets.

### 3.3. Drug-compound-target-disease network construction and analysis

**Figure 3.**
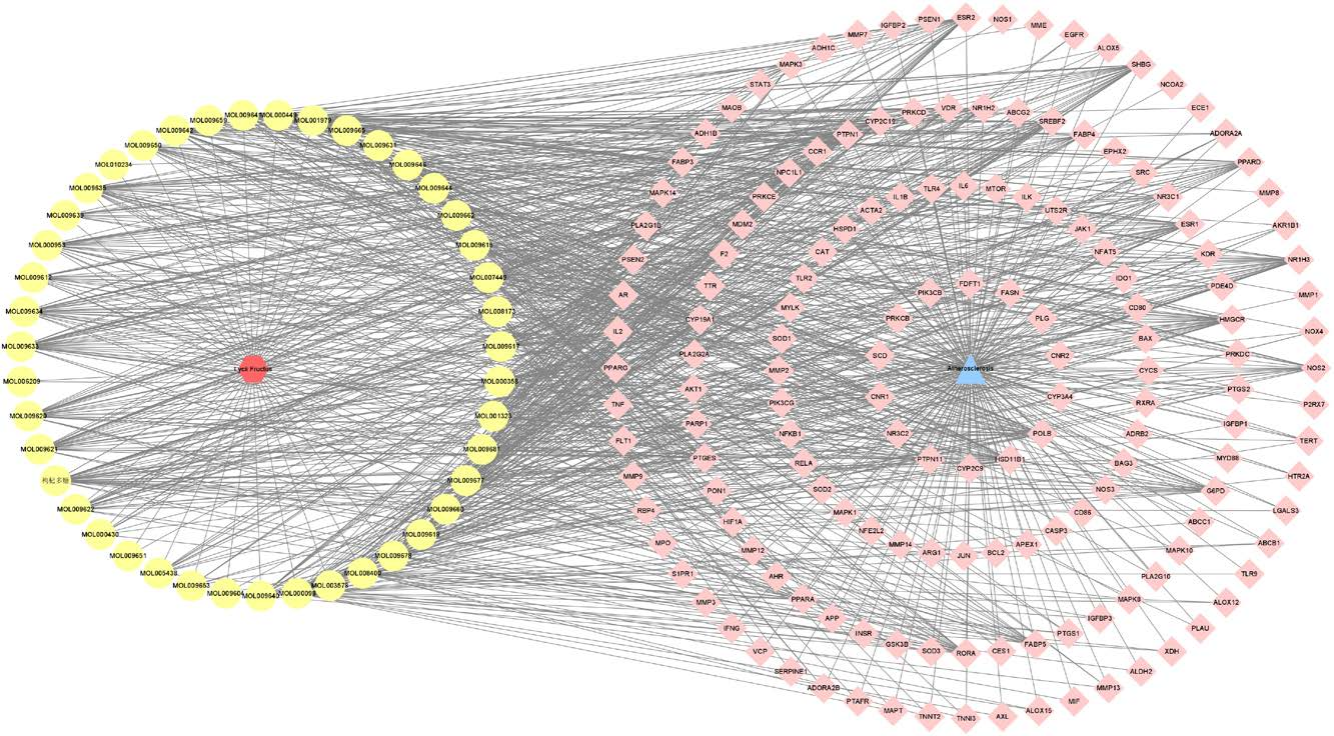
The network of active compounds of Goji berries and AS targeting proteins. The red hexagon represents Goji berries, the yellow circle represents 44 active compounds, the blue triangle represents atherosclerosis, and the pink quadrilateral represents 159 targeted proteins.

The above-obtained targets for each compound of Goji berries and AS-related targets were converted to standard gene names through the Uniport database. The data related to the 44 major compounds of Goji berries and its 159 action targets in AS were entered into Excel software for collation and imported into Cytoscape software to obtain the drug-compound-target-disease network diagram of Goji berries (Fig. 3). The red hexagon in the figure represents Goji berries, the connected yellow circles represent the 44 active compounds, the blue triangle represents AS, and the pink quadrilateral it is connected to represents 159 action targets. Each edge within the diagram signifies a connection, which shows that 1 herb corresponds to multiple compounds, 1 compound corresponds to multiple targets, and conversely, 1 target also corresponds to multiple compounds, and 1 compound may also correspond to multiple herbs, which intuitively shows the characteristics of multi-compound and multi-target of herb medicine.

### 3.4. PPI network construction

**Figure 4.**
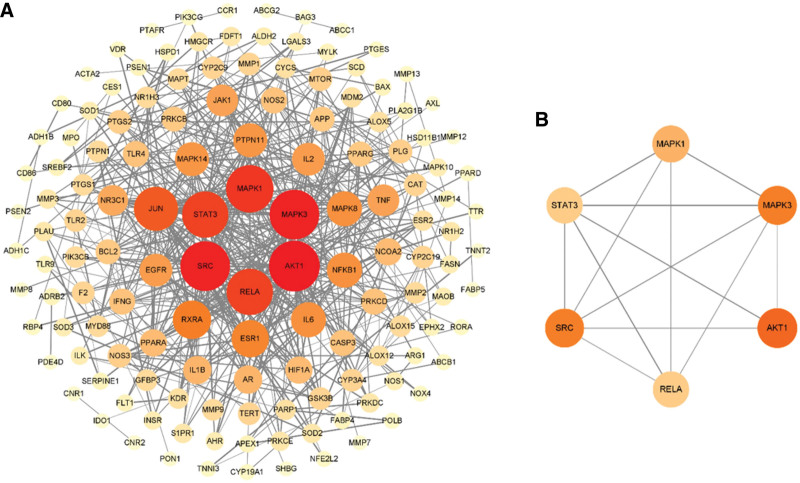
Protein-protein interaction (PPI) network of the target proteins common to Goji berries and AS. (A) PPI network, nodes represent proteins, and the edges stand for the relationships. (B) The core proteins from the PPI network (sorted by the “degree” values).

The 159 potential targets of Goji berries in AS were uploaded to the STRING platform with the species set to *Homo sapiens* and the condition set to “highest confidence ≥0.9” to obtain the PPI network. The TSV file was downloaded and imported into Cytoscape software for network topology analysis (Fig. 4A). The size and color of nodes reflect the Degree of nodes, and the thickness of edges reflects the Combined score between nodes. There are 139 targets and 504 edges in Figure 4A. Based on the Degree value of the PPI network, the top 6 core target proteins were AKT1, SRC, MAPK3, MAPK1, RELA, and STAT3 (Fig. 4B).

### 3.5. GO biological process and KEGG pathway analysis

**Figure 5.**
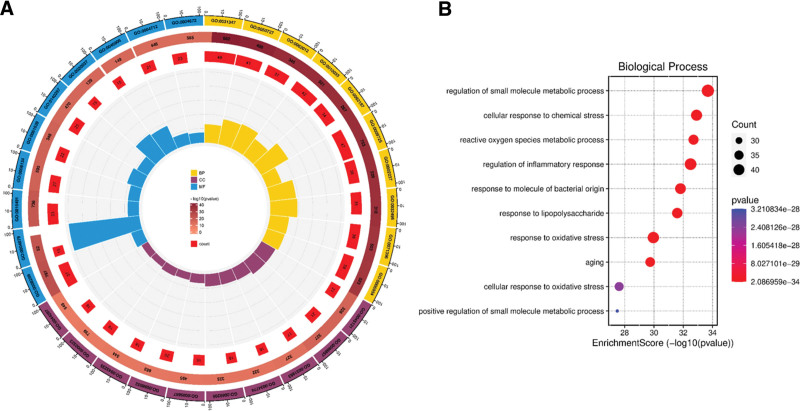
Gene ontology (GO) analysis of the target proteins common to Goji berries and atherosclerosis (AS). (A) The outermost layer displays the names of GO analysis entries. The second layer reflects the gene count, with color intensity indicating significance. The third layer represents the count of genes. The innermost column size signifies the level of enrichment. BP = biological process, CC = cellular component, and MF = molecular function. (B) GO analysis of the first ten entries of biological processes, larger bubbles mean more genes are enriched, and redder color means smaller *P* value.

**Figure 6.**
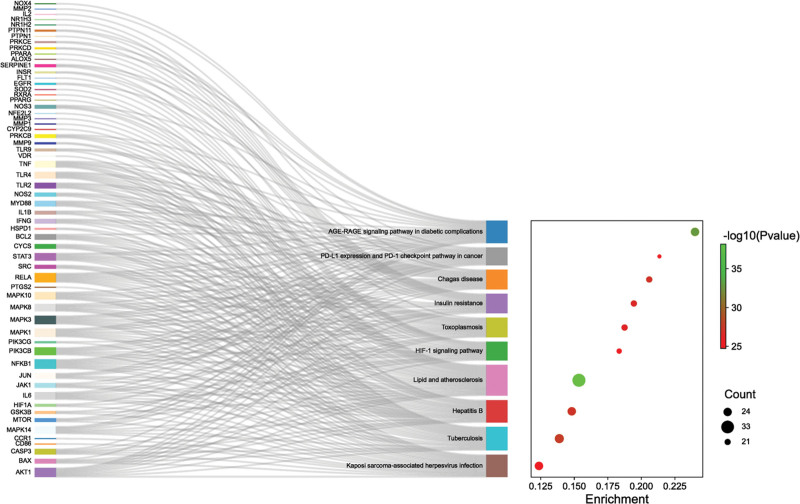
Kyoto encyclopedia of genes and genomes (KEGG) analysis of the target proteins common to Goji berries and atherosclerosis (AS). The top 10 entries were shown in the middle. The size of the nodes on the left represents the more edges connected. Larger bubbles on the right indicate more enriched genes.

**Figure 7.**
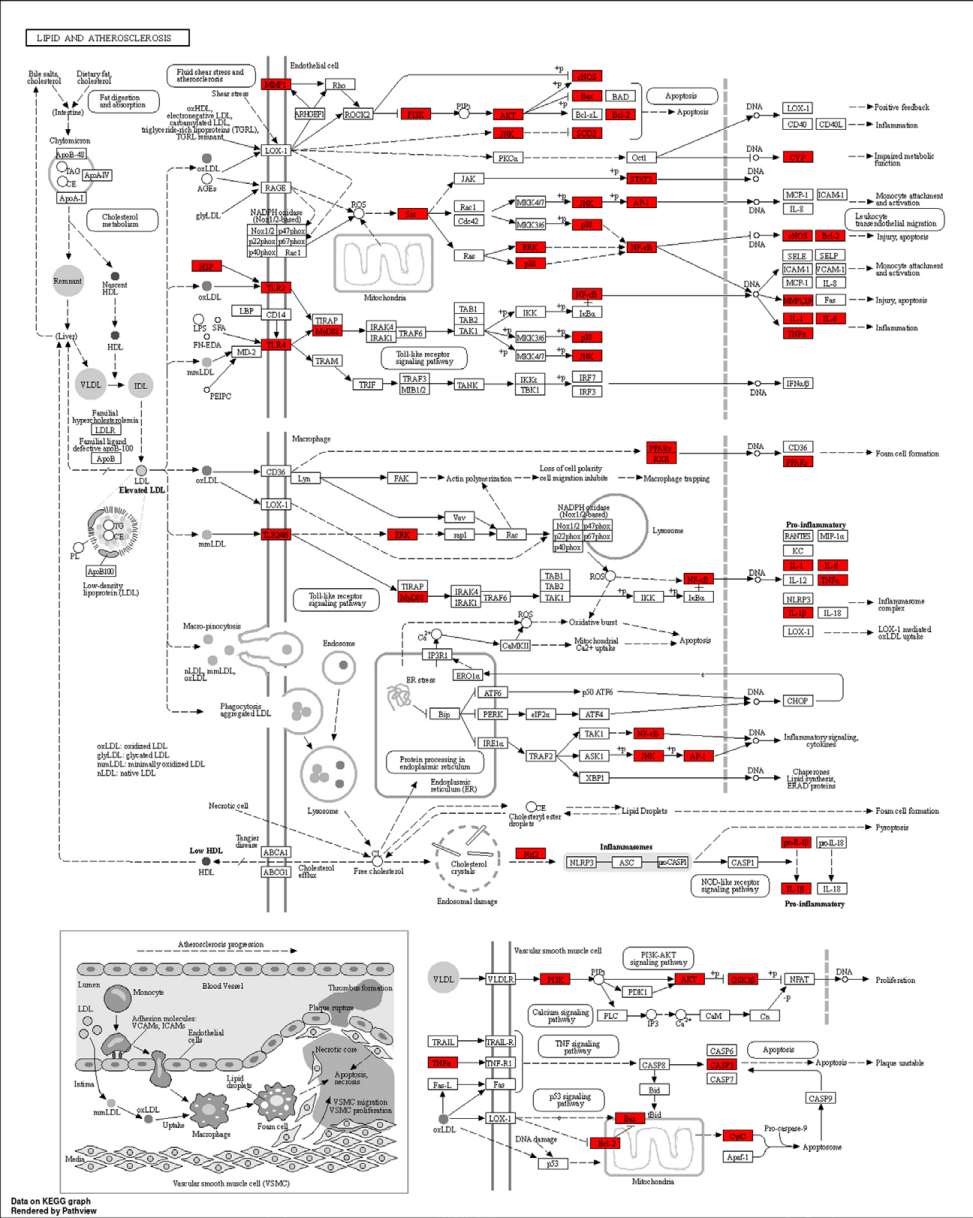
Pathway map of Goji berries in lipids and atherosclerosis (AS). Target proteins analyzed in this study are highlighted in red.

To better understand the mechanism pathways by which the potentially acting proteins of Goji berries play a role in AS, we imported the 159 potential targets obtained from the above analysis into the Metascape database, selected the species as *Homo sapiens*, and performed GO biological process (BP) and KEGG pathway enrichment analysis, respectively. The top ten entries for GO BP, cellular component, and molecular function (MF) were visualized (Fig. 5A). BP include regulation of small molecule metabolic processes, cellular responses to chemical stress, reactive oxygen metabolic processes, and regulation of inflammatory responses (Fig. 5B). The top ten KEGG pathways, including lipid and AS, AGE-RAGE signaling pathway, Chagas disease, and insulin resistance, were chosen for visualization by Sankey diagram (Fig. 6). Notably, lipids and AS are the main pathways we are interested in, which contain several identified core targets such as AKT1, MAPK, SRC, STAT3, etc. (Fig. 7).

### 3.6. Molecular docking assessment

**Figure 8.**
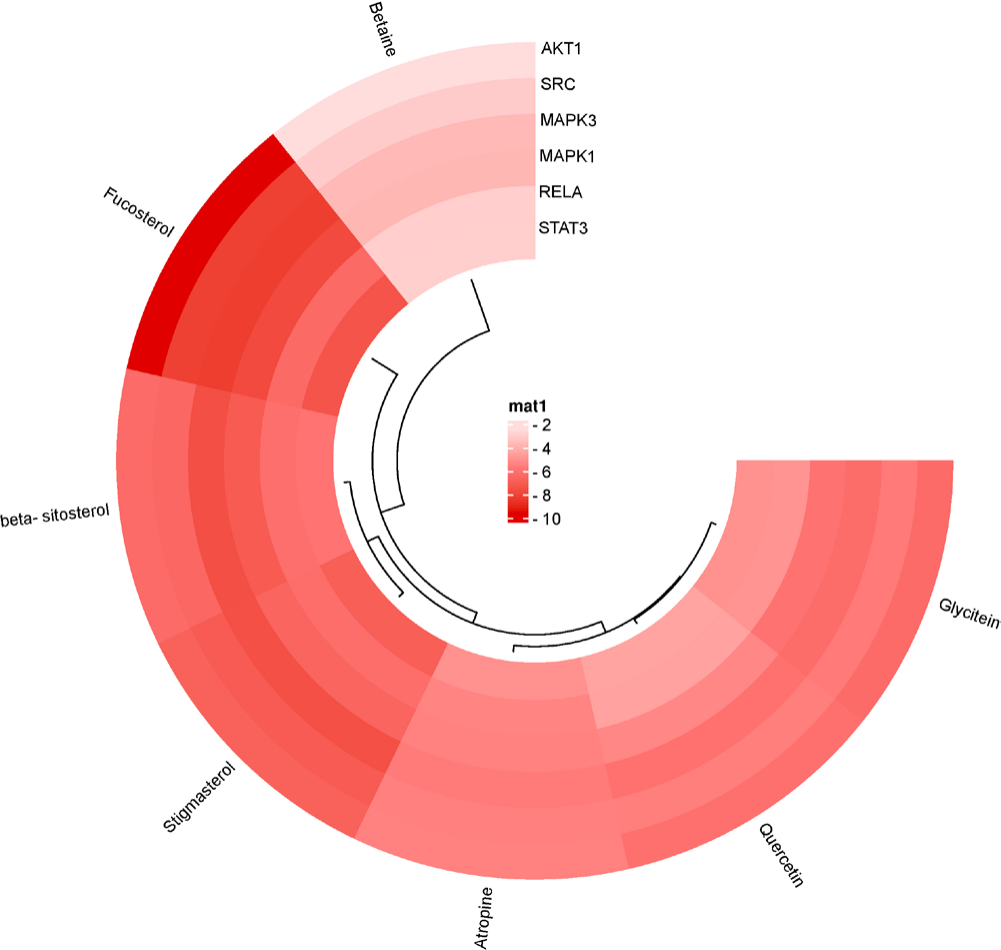
Heat map of molecular docking. Redder color means lower binding energy.

**Figure 9.**
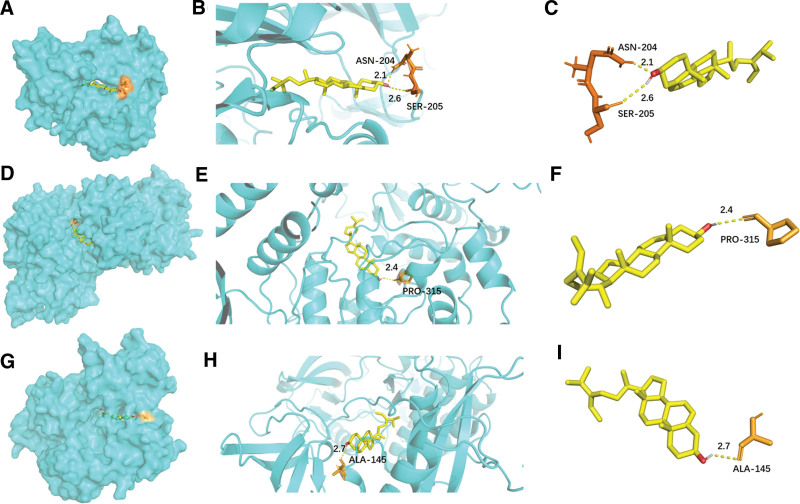
Molecular docking between fucosterol and the top core proteins (AKT1, MAPK3, and SRC). (A–C) Fucosterol-AKT1. (D–F) Fucosterol-MAPK3. (G–I) Fucosterol-SRC.

The 6 core target proteins of Goji berries acting on AS: AKT1, SRC, MAPK3, MAPK1, RELA, and STAT3, which were obtained in PPI network analysis, were molecularly docked with the 7 critical components of Goji berries: Atropine, betaine, β-sitosterol, quercetin, glycitein, fucosterol and stigmasterol. It is generally accepted that smaller binding energies indicate stronger interactions and heightened stability within the binding conformation of ligands to receptors (Table 2). The lighter color in the thermogram represents the lower binding energy (Fig. 8). Notably, molecular docking analysis revealed fucosterol potential to bind to AKT1 through the formation of hydrogen bonds via ASN-204 (length: 2.1 Å), SER-205 (2.6 Å) (Fig. 9A–C). Additionally, fucosterol is also predicted to dock into the binding pocket of MAPK3 via a single hydrogen bond with PRO-315 at a distance of 2.4 Å (Fig. 9D–F). Lastly, fucosterol and SRC bind via a hydrogen bond formed at ALA-145 (2.7 Å) (Fig. 9G–I).

**Table 2:** Binding energy of 7 active compounds of Goji berries and 6 core proteins.

Ligand	Binding energy (kcal/mol)					
	AKT1	SRC	MAPK3	MAPK1	RELA	STAT3
Atropine	−5.57	−5.59	−5.75	−5.53	−5.46	−5.07
Betaine	−2.54	−3.14	−3.75	−3.83	−3.05	−3.03
beta-sitosterol	−6.42	−6.58	−7.65	−7.15	−6.23	−5.99
Quercetin	−6.17	−5.63	−6.12	−5.33	−4.51	−4.45
Glycitein	−6.38	−5.77	−6.35	−6.01	−4.95	−5.07
Fucosterol	−9.68	−8.24	−8.32	−7.85	−6.44	−7.44
Stigmasterol	−6.81	−7.12	−7.61	−6.67	−6.27	−6.97

## 4. Discussion

CVD is a widespread challenge in contemporary society. AS occurring in the coronary and cerebral arteries is the predominant instigator of CVD with a substantial population-level mortality burden. AS is characterized by the gradual accumulation of lipids and inflammatory cells within the aortic Intima.^[29]^ AS alone is rarely fatal, thrombosis coupled with plaque rupture can greatly increase mortality.^[30]^ With the improvement of modern quality of life, people are more prone to consuming high-calorie food, leading to an increase in the obesity rate among the population. Obesity triggers the aberrant secretion of adipose tissue-derived cytokines, such as C-reactive protein, adiponectin, leptin, vascular endothelial growth factor, TNFα, IL-6, etc. This abnormal cytokine profile can accelerate endothelial inflammatory responses. The closely associated disturbances in lipid metabolism, abnormal blood rheology, insulin resistance, and other factors related to obesity can also induce endothelial dysfunction. These multifaceted factors collectively expedite the progression of AS.^[31]^ Therefore, it is important to study the treatment and prevention of atherosclerotic disease.

In this study, we explored Goji berries potential mechanisms for treating AS using network pharmacology and molecular docking assessment. In the current landscape of clinical research on Goji berries treatment for AS, it has been observed that elderly individuals afflicted with AS experience a substantial alleviation of clinical symptoms after a 15-day of oral administration of Goji berries-based TCM formulations.^[32]^ Similarly, elderly males with hyperlipidemia who consume daily water extracts of Goji berries exhibit effective reductions in serum levels of total cholesterol, triglycerides, high-density lipoprotein cholesterol, and low-density lipoprotein cholesterol.^[33]^ The critical active compounds of Goji berries include atropine, betaine, β-sitosterol, quercetin, glycitein, fucosterol, stigmasterol etc. Among them, β-sitosterol, stigmasterol, and fucosterol belong to phytosterols, and phytosterols can reduce intestinal absorption of dietary and biliary cholesterol by 30% to 50%.^[34]^

Additionally, there is evidence that 2 g of phytosterols per day significantly inhibits cholesterol absorption and reduces low-density lipoprotein cholesterol levels by 8% to 10%.^[35]^ Stigmasterol cholesterol efflux macrophages and inhibits the secretion of the inflammatory cytokines TNFα, IL-6, and IL-1β.^[36]^ In addition to phytosterols, quercetin can inhibit inflammation and apoptosis through the reactive oxygen species-regulated PI3K/AKT signaling pathway.^[37]^ Moreover, quercetin ameliorates lipid deposition and overproduction of reactive oxygen species.^[38]^ Several studies have shown that quercetin has significant anti-inflammatory and antioxidant effects,^[39–41]^ and can be used as a potential drug for preventing and treating AS. Betaine inhibits the NF-κB inflammatory signaling pathway,^[42]^ and as a dietary supplement, reduces the development of AS in apoE-deficient mice.^[43]^ Glycitein significantly reduces plasma total cholesterol and non-high-density lipoprotein cholesterol. Its heightened bioavailability underscores its potent cholesterol-lowering efficacy.^[44]^ Notably, glycitein has a therapeutic effect on AS by restraining the proliferation of aortic smooth muscle cells in stroke-prone spontaneously hypertensive rats.^[45]^ In addition, *Lycium barbarum* polysaccharide, which is rich in Goji berries, can inhibit LPS-induced inflammation,^[46]^ has anti-obesity activity,^[47]^ and is beneficial for chronic metabolic diseases associated with diabetes and high-fat diet. AKT1, SRC, MAPK3, MAPK1, RELA, and STAT3 are the core target proteins in the PPI network (Table 2 and Fig. 4). In a mouse model of AS, knockout of AKT1 in vivo increases the burden of atherosclerotic lesions and promotes coronary AS.^[48]^ In addition, activation of AKT1 stands as a regulatory factor in AS.^[49]^ Inflammation has been shown to play an important role in the development and progression of atherosclerotic plaques. NF-κB transcription factor family, including c-JUN and RELA belong to the NF-κB transcription factor family,^[50]^ partake in the activation of the NF-κB pathway, playing a central role in inflammation and is a crucial regulator of inflammation and cell death in the pathogenesis of AS.^[51]^ Meanwhile, activation of SRC and NF-κB enhances the expression of interleukin IL-6 and increases the nuclear translocation of c-JUN and p65,^[52]^ suggesting a role for SRC in the inflammatory response. The interplay with the STAT3 pathway closely corresponds to the IL-6 cytokine family,^[53]^ which plays an important role in endothelial cell dysfunction during AS. Recently, a growing body of evidence suggests that targeted inhibition of STAT3 could be a potential therapeutic strategy for AS.^[54]^ Our analysis suggests multiple core targets involved in the classical inflammatory pathways of AS.

KEGG analysis showed that the signaling pathways associated with AS include lipids and AS, AGE-RAGE (Advanced Glycation End-product-Receptor for Advanced Glycation End-product) signaling pathway, insulin resistance, and IL-17 signaling pathway. The hallmark lesion characteristic of AS resides in lipid deposition within the arterial portion. In its early stages, atherosclerotic development is called “fatty streaks,” which are areas of intracellular lipid accumulation in the vessel wall characterized by foam cells. Notably, diabetes and AS are inextricably linked.^[55–57]^ Advanced glycation end-product (AGEs) accumulate in diabetic patients and appear to play an important role in the development of AS. AGEs are involved in the atherosclerotic process either directly or through receptor-mediated mechanisms, and the most widely studied receptor is RAGE (receptor for AGEs).^[58]^ RAGE deficiency reduces atherosclerotic plaque area in diabetic mice.^[58]^ Indubitably, insulin resistance directly contributes to the development of atherosclerotic CVD by inhibiting nitric oxide production (endothelial dysfunction) and stimulating MAPK pathways.^[59,60]^ Our analysis showed multiple targets of Goji berries involved in atherosclerotic pathways (Fig. 7), demonstrating that Goji berries can be used as a potential drug for the treatment of AS.

Molecular docking assessment showed that the phytosterol, β-sitosterol, stigmasterol, and fucosterol have high binding energy to all core target proteins (Table 2). Furthermore, other compounds of Goji berries have also demonstrated favorable docking outcomes, thereby underscoring their promise as active compounds harboring therapeutic potential against AS. The above results reflect that Goji berries have a multi-component, multi-target, and multi-pathway biological property. These intricate elements, spanning compounds, targets, and pathways, intricately interlace into a comprehensive network, embodying a holistic approach to AS treatment.

It should be noted that this study has certain limitations. Firstly, the data on bioactive compounds and targets were retrieved from literature and databases. Hence, the reliability and accuracy of predictions are contingent upon the quality of the data. Secondly, this research employed data mining techniques; further cellular experiments, animal studies, and clinical trials are required to validate the findings.

## 5. Conclusions

Quercetin, betaine, atropine, β-sitosterol, fucosterol, and stigmasterol may be the main active compounds of Goji berries for the treatment of AS. Its critical targets include AKT1, SRC, MAPK1, MAPK3, RELA, and STAT3. The main indicated pathways are lipid and AS and the AGE-RAGE signaling pathway in diabetic complications. The therapeutic effects of Goji berries in treating AS might be mediated through multi-compounds, multi-targets, and multi-pathways, which required further research for validation.

## Acknowledgments

We thank Dr Haijie Yu and Dr Xinzhi Li for the fruitful discussion.

## Author contributions

**Conceptualization:** Xinchen Qin, Zikai Xie, Xi Chen, Xiaoxuan Wang.

**Data curation:** Xinchen Qin, Zikai Xie, Xiaoxuan Wang.

**Investigation:** Lijuan Ma.

**Methodology:** Xinchen Qin, Zikai Xie, Xi Chen.

**Supervision:** Lijuan Ma.

**Writing – original draft:** Xinchen Qin.

**Writing – review & editing:** Lijuan Ma.
